# Diaqua­dichloridobis(pyridine-κ*N*)manganese(II)

**DOI:** 10.1107/S160053681103546X

**Published:** 2011-09-14

**Authors:** M. Karthikeyan, S. Karthikeyan, Bala. Manimaran

**Affiliations:** a Department of Chemistry, Pondicherry University, Puducherry 605 014, India

## Abstract

The molecular title compound, [MnCl_2_(C_5_H_5_N)_2_(H_2_O)_2_], lies about an inversion centre. The Mn^II^ atom is in an all-*trans* octa­hedral environment defined by two water mol­ecules, two chloride anions and two pyridine ligands. An inter­molecular hydrogen-bonding inter­action between a water mol­ecule and a chloride anion bonded to an adjacent Mn^II^ atom generates an eight-membered ring. The crystal packing exhibits two inter­molecular π–π stacking inter­actions between the aromatic rings, with centroid–centroid distances of 3.485 (12) and 3.532 (12) Å.

## Related literature

For hydrogen-bond motifs, see: Frost *et al.* (2006 ▶). For related structures, see: Cotton *et al.* (1995 ▶); Kruszynski *et al.* (2001 ▶).
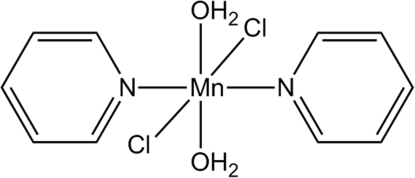


## Experimental

### 

#### Crystal data

[MnCl_2_(C_5_H_5_N)_2_(H_2_O)_2_]
*M* *_r_* = 320.07
Triclinic, 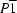

*a* = 6.2290 (5) Å
*b* = 6.6327 (5) Å
*c* = 8.6831 (7) Å
α = 108.931 (7)°
β = 103.499 (7)°
γ = 96.969 (6)°
*V* = 322.30 (4) Å^3^
*Z* = 1
Mo *K*α radiation
μ = 1.43 mm^−1^
*T* = 150 K
0.26 × 0.14 × 0.07 mm

#### Data collection

Oxford Diffraction Xcalibur Eos diffractometer
Absorption correction: multi-scan (*CrysAlis PRO*; Oxford Diffraction, 2009 ▶) *T*_min_ = 0.733, *T*_max_ = 1.000
1951 measured reflections
1137 independent reflections
1031 reflections with *I* > 2σ(*I*)
*R*_int_ = 0.022

#### Refinement

*R*[*F* ^2^ > 2σ(*F* ^2^)] = 0.022
*wR*(*F* ^2^) = 0.056
*S* = 1.05
1137 reflections
87 parameters
2 restraints
H atoms treated by a mixture of independent and constrained refinement
Δρ_max_ = 0.27 e Å^−3^
Δρ_min_ = −0.22 e Å^−3^

### 

Data collection: *CrysAlis CCD* (Oxford Diffraction, 2009 ▶); cell refinement: *CrysAlis RED* (Oxford Diffraction, 2009 ▶); data reduction: *CrysAlis RED*; program(s) used to solve structure: *SHELXS97* (Sheldrick, 2008 ▶); program(s) used to refine structure: *SHELXL97* (Sheldrick, 2008 ▶); molecular graphics: *ORTEP-3 for Windows* (Farrugia, 1997 ▶) and *PLATON* (Spek, 2009 ▶); software used to prepare material for publication: *PLATON*.

## Supplementary Material

Crystal structure: contains datablock(s) I, global. DOI: 10.1107/S160053681103546X/ng5215sup1.cif

Structure factors: contains datablock(s) I. DOI: 10.1107/S160053681103546X/ng5215Isup2.hkl

Additional supplementary materials:  crystallographic information; 3D view; checkCIF report

## Figures and Tables

**Table 1:** Hydrogen-bond geometry (Å, °)

*D*—H⋯*A*	*D*—H	H⋯*A*	*D*⋯*A*	*D*—H⋯*A*
O1—H1*B*⋯Cl1^i^	0.86 (1)	2.38 (1)	3.2301 (13)	170 (2)

Symmetry code: (i) 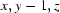.

**Fig. 1.**
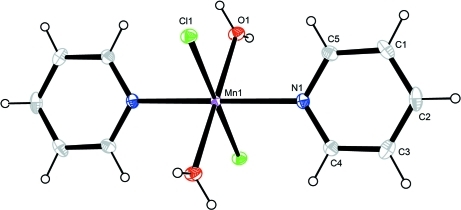
: The molecular structure of title compound, showing displacement ellipsoids drawn at the 50% probability level.

**Fig. 2.**
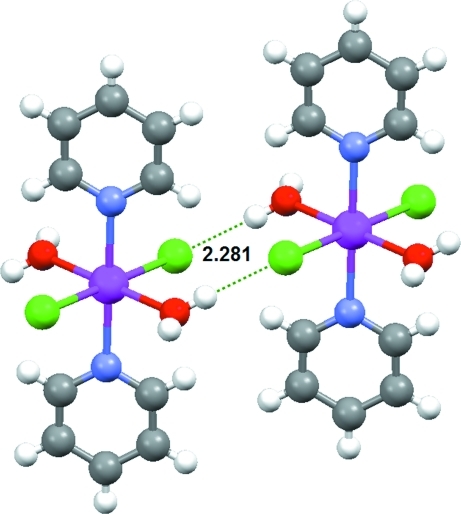
: A view of the intermolecular (O1—H1A···Cl1) hydrogen bonding interaction shown as dashed line.

**Fig. 3.**
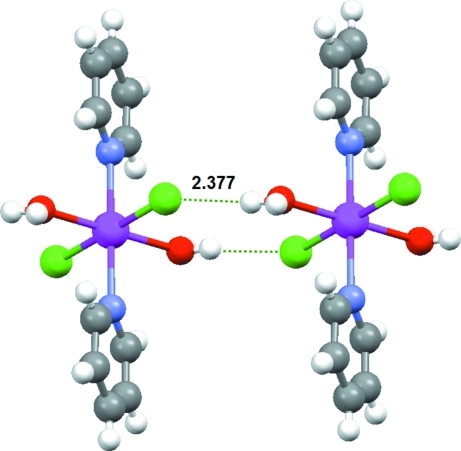
: A view of the intermolecular (O1—H1B···Cl1) hydrogen bonding interaction shown as dashed line.

**Fig. 4.**
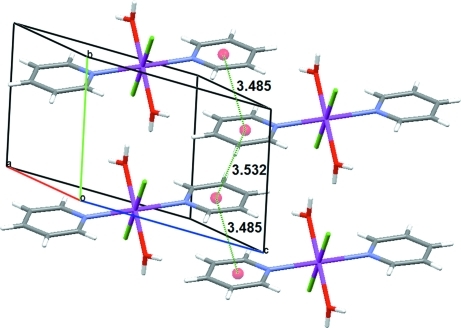
: A view of the crystal packing showing intermolecular π···π stacking interaction.

## supplementary crystallographic information

### Comment

The molecular structure of the title compound C_10_H_14_Cl_2_MnN_2_O_2_ is isostructural with Cr (Cotton *et al.*, 1995) compound. The title compound is shown in Fig. 1. The bond length of Mn—N, Mn—O, and Mn—Cl is comparable to those observed in a related crystal structure namely that diaqua-dichloro-bis(1-((2-(2,4-dichlorophenyl)-1,3-dioxalan-2-yl)methyl)-4*H*-1,2,4-triazol-4-yl)-manganese(II) (Kruszynski *et al.*, 2001). The molecule exibits two intermolecular hydrogen bonding (O—H···Cl) between water molecule coordinated to manganese and a chloride bonded to adjacent manganese center with a distance of 2.281 (15) Å (Fig. 2) and 2.377 (16) Å (Fig. 3) which leads to layered structure (Frost *et al.*., 2006). The crystal packing exhibits two intermolecular π–π stacking interaction between the aromatic rings with the centroid to centroid distance of 3.485 (12) and 3.532 (12) Å (Fig. 4).

### Experimental

A solution of manganese(II) chloride tetrahydrate (692 mg, 3.5 mmol) in distilled water (2 ml) was added a solution of pyridine (0.28 ml, 3.5 mmol) in a 1:1 ethanol-water mixture (2 ml) slowly. Colorless white crystals began to form at ambient temperature after a week.

### Refinement

The non-hydrogen atoms were refined anisotropically whereas hydrogen atoms were refined isotropically. The other hydrogen atoms were placed in calculated positions (C—H = 0.93 Å) and included in the refinement in a riding-model approximation with *U*_iso_(H) =1.2U_eq_(C). The water H atoms were refined with a distance restraint.

### Crystal data

**Table:**

[MnCl_2_(C_5_H_5_N)_2_(H_2_O)_2_]	*Z* = 1
*M**_r_* = 320.07	*F*(000) = 163
Triclinic, *P*1	*D*_x_ = 1.649 Mg m^−^^3^
Hall symbol: -P 1	Mo *K*α radiation, λ = 0.71073 Å
*a* = 6.2290 (5) Å	Cell parameters from 1883 reflections
*b* = 6.6327 (5) Å	θ = 2.6–28.8°
*c* = 8.6831 (7) Å	µ = 1.43 mm^−^^1^
α = 108.931 (7)°	*T* = 150 K
β = 103.499 (7)°	Block, colorless
γ = 96.969 (6)°	0.26 × 0.14 × 0.07 mm
*V* = 322.30 (4) Å^3^	

### Data collection

**Table:**

Oxford Diffraction Xcalibur Eos diffractometer	1137 independent reflections
Radiation source: fine-focus sealed tube	1031 reflections with *I* > 2σ(*I*)
graphite	*R*_int_ = 0.022
Detector resolution: 15.9821 pixels mm^-1^	θ_max_ = 25.0°, θ_min_ = 2.6°
ω scans	*h* = −7→5
Absorption correction: multi-scan (*CrysAlis PRO*; Oxford Diffraction, 2009)	*k* = −7→7
*T*_min_ = 0.733, *T*_max_ = 1.000	*l* = −10→10
1951 measured reflections	

### Refinement

**Table:**

Refinement on *F*^2^	Primary atom site location: structure-invariant direct methods
Least-squares matrix: full	Secondary atom site location: difference Fourier map
*R*[*F*^2^ > 2σ(*F*^2^)] = 0.022	Hydrogen site location: inferred from neighbouring sites
*wR*(*F*^2^) = 0.056	H atoms treated by a mixture of independent and constrained refinement
*S* = 1.05	*w* = 1/[σ^2^(*F*_o_^2^) + (0.0294*P*)^2^ + 0.1426*P*] where *P* = (*F*_o_^2^ + 2*F*_c_^2^)/3
1137 reflections	(Δ/σ)_max_ = 0.011
87 parameters	Δρ_max_ = 0.27 e Å^−^^3^
2 restraints	Δρ_min_ = −0.22 e Å^−^^3^

### Special details

**Table:**

Geometry. All e.s.d.'s (except the e.s.d. in the dihedral angle between two l.s. planes) are estimated using the full covariance matrix. The cell e.s.d.'s are taken into account individually in the estimation of e.s.d.'s in distances, angles and torsion angles; correlations between e.s.d.'s in cell parameters are only used when they are defined by crystal symmetry. An approximate (isotropic) treatment of cell e.s.d.'s is used for estimating e.s.d.'s involving l.s. planes.
Refinement. Refinement of *F*^2^ against ALL reflections. The weighted *R*-factor *wR* and goodness of fit *S* are based on *F*^2^, conventional *R*-factors *R* are based on *F*, with *F* set to zero for negative *F*^2^. The threshold expression of *F*^2^ > σ(*F*^2^) is used only for calculating *R*-factors(gt) *etc*. and is not relevant to the choice of reflections for refinement. *R*-factors based on *F*^2^ are statistically about twice as large as those based on *F*, and *R*- factors based on ALL data will be even larger.

### Fractional atomic coordinates and isotropic or equivalent isotropic displacement parameters (Å^2^)

**Table:**

	*x*	*y*	*z*	*U*_iso_*/*U*_eq_	
H1A	0.097 (2)	−0.252 (4)	0.484 (3)	0.038 (7)*	
H1B	0.237 (5)	−0.3957 (19)	0.455 (3)	0.045 (8)*	
Mn1	0.5000	0.0000	0.5000	0.00900 (13)	
Cl1	0.22317 (7)	0.23567 (7)	0.43191 (5)	0.01375 (13)	
N1	0.4713 (2)	−0.1480 (2)	0.22133 (17)	0.0115 (3)	
O1	0.2181 (2)	−0.2694 (2)	0.45397 (16)	0.0150 (3)	
C3	0.6365 (3)	−0.2269 (3)	−0.0085 (2)	0.0169 (4)	
H3	0.7657	−0.2195	−0.0444	0.020*	
C2	0.4261 (3)	−0.3200 (3)	−0.1260 (2)	0.0186 (4)	
H2	0.4112	−0.3782	−0.2419	0.022*	
C4	0.6529 (3)	−0.1449 (3)	0.1626 (2)	0.0139 (4)	
H4	0.7956	−0.0850	0.2407	0.017*	
C5	0.2673 (3)	−0.2372 (3)	0.1057 (2)	0.0147 (4)	
H5	0.1398	−0.2401	0.1440	0.018*	
C1	0.2383 (3)	−0.3246 (3)	−0.0671 (2)	0.0188 (4)	
H1	0.0943	−0.3857	−0.1429	0.023*	

### Atomic displacement parameters (Å^2^)

**Table:**

	*U*^11^	*U*^22^	*U*^33^	*U*^12^	*U*^13^	*U*^23^
Mn1	0.0090 (2)	0.0087 (2)	0.0082 (2)	0.00120 (14)	0.00223 (14)	0.00214 (15)
Cl1	0.0119 (2)	0.0125 (2)	0.0175 (2)	0.00426 (17)	0.00427 (18)	0.00570 (18)
N1	0.0138 (8)	0.0098 (7)	0.0107 (7)	0.0030 (6)	0.0030 (6)	0.0037 (6)
O1	0.0135 (7)	0.0115 (7)	0.0213 (7)	0.0018 (5)	0.0070 (5)	0.0066 (6)
C3	0.0228 (10)	0.0162 (10)	0.0186 (9)	0.0092 (8)	0.0120 (8)	0.0092 (8)
C2	0.0336 (11)	0.0135 (9)	0.0102 (8)	0.0087 (8)	0.0064 (8)	0.0051 (7)
C4	0.0144 (9)	0.0114 (9)	0.0151 (9)	0.0038 (7)	0.0030 (7)	0.0043 (7)
C5	0.0139 (9)	0.0140 (9)	0.0154 (9)	0.0011 (7)	0.0028 (7)	0.0060 (7)
C1	0.0207 (10)	0.0148 (10)	0.0138 (9)	0.0010 (8)	−0.0030 (8)	0.0031 (8)

### Geometric parameters (Å, °)

**Table:**

Mn1—O1^i^	2.2100 (13)	C3—C2	1.382 (3)
Mn1—O1	2.2100 (13)	C3—C4	1.381 (2)
Mn1—N1	2.2505 (14)	C3—H3	0.9300
Mn1—N1^i^	2.2505 (14)	C2—C1	1.383 (3)
Mn1—Cl1	2.5582 (4)	C2—H2	0.9300
Mn1—Cl1^i^	2.5582 (4)	C4—H4	0.9300
N1—C4	1.346 (2)	C5—C1	1.379 (3)
N1—C5	1.345 (2)	C5—H5	0.9300
O1—H1A	0.8629 (10)	C1—H1	0.9300
O1—H1B	0.8630 (10)		
			
O1^i^—Mn1—O1	180.00 (5)	Mn1—O1—H1A	124.5 (16)
O1^i^—Mn1—N1	92.86 (5)	Mn1—O1—H1B	123.3 (18)
O1—Mn1—N1	87.14 (5)	H1A—O1—H1B	105 (2)
O1^i^—Mn1—N1^i^	87.14 (5)	C2—C3—C4	119.36 (17)
O1—Mn1—N1^i^	92.86 (5)	C2—C3—H3	120.3
N1—Mn1—N1^i^	180.00 (3)	C4—C3—H3	120.3
O1^i^—Mn1—Cl1	88.86 (3)	C3—C2—C1	118.34 (17)
O1—Mn1—Cl1	91.14 (3)	C3—C2—H2	120.8
N1—Mn1—Cl1	89.26 (4)	C1—C2—H2	120.8
N1^i^—Mn1—Cl1	90.74 (4)	N1—C4—C3	122.77 (17)
O1^i^—Mn1—Cl1^i^	91.14 (3)	N1—C4—H4	118.6
O1—Mn1—Cl1^i^	88.86 (3)	C3—C4—H4	118.6
N1—Mn1—Cl1^i^	90.74 (4)	N1—C5—C1	123.08 (17)
N1^i^—Mn1—Cl1^i^	89.26 (4)	N1—C5—H5	118.5
Cl1—Mn1—Cl1^i^	180.0	C1—C5—H5	118.5
C4—N1—C5	117.32 (15)	C5—C1—C2	119.12 (17)
C4—N1—Mn1	122.28 (11)	C5—C1—H1	120.4
C5—N1—Mn1	120.36 (12)	C2—C1—H1	120.4
			
O1^i^—Mn1—N1—C4	35.44 (13)	Cl1^i^—Mn1—N1—C5	126.61 (12)
O1—Mn1—N1—C4	−144.56 (13)	C4—C3—C2—C1	0.9 (3)
N1^i^—Mn1—N1—C4	174 (7)	C5—N1—C4—C3	0.5 (2)
Cl1—Mn1—N1—C4	124.26 (13)	Mn1—N1—C4—C3	−177.19 (13)
Cl1^i^—Mn1—N1—C4	−55.74 (13)	C2—C3—C4—N1	−1.2 (3)
O1^i^—Mn1—N1—C5	−142.21 (13)	C4—N1—C5—C1	0.3 (3)
O1—Mn1—N1—C5	37.79 (13)	Mn1—N1—C5—C1	178.05 (14)
N1^i^—Mn1—N1—C5	−4(7)	N1—C5—C1—C2	−0.5 (3)
Cl1—Mn1—N1—C5	−53.39 (12)	C3—C2—C1—C5	−0.2 (3)

Symmetry codes: (i) −*x*+1, −*y*, −*z*+1.

### Hydrogen-bond geometry (Å, °)

**Table:**

*D*—H···*A*	*D*—H	H···*A*	*D*···*A*	*D*—H···*A*
O1—H1B···Cl1^ii^	0.86 (1)	2.38 (1)	3.2301 (13)	170 (2)

Symmetry codes: (ii) *x*, *y*−1, *z*.
